# Field epidemiology training in Tunisia: achievements and gaps

**DOI:** 10.3389/fpubh.2026.1863895

**Published:** 2026-08-19

**Authors:** Nejib Charaa, Hajer Letaief, Mohamed Kouni Chahed, Nissaf Ben Alaya

**Affiliations:** 1 National Observatory of New and Emerging Diseases, Ministry of Health, Tunis, Tunisia; 2 Faculty of Medicine of Tunis, University of Tunis el Manar, Tunis, Tunisia

**Keywords:** applied epidemiology, disease surveillance, epidemiology training, field epidemiology, field epidemiology training programs, outbreak response, public health education, public health workforce

## Abstract

**Introduction:**

National health security depends on a trained field epidemiology workforce. This study evaluates the Tunisia Field Epidemiology Training Program (T-FETP) from its launch in 2018 through 2024, with a focus on workforce capacity, geographic coverage, and structural barriers to program sustainability.

**Methods:**

We conducted a descriptive cross-sectional analysis of the national T-FETP graduate roster. Territorial coverage was assessed against the international benchmark of one Intermediate-level FETP graduate per 200,000 inhabitants. Density and capacity gaps were analyzed for each of Tunisia’s 24 governorates.

**Results:**

A total of 103 professionals were trained across seven cohorts, including 50 at the Intermediate level. While national density reached 0.83 per 200,000 inhabitants, it decreased to 0.57 when restricted to regional and local levels. Only 25% of governorates met the recommended threshold, and six had no Intermediate-level epidemiologist. The estimated cumulative national shortfall was 43 professionals. Marked geographic disparities were observed, with lower coverage in densely populated areas. Among graduates, 75.7% were female and 70.5% were physicians. In 2026, the median age of Intermediate graduates was 54 years.

**Conclusion:**

Expanding training output alone has not produced adequate or equitable field epidemiology capacity in Tunisia. Geographic disparities, limited institutionalization, and the absence of sustainable domestic financing remain key constraints. Addressing these gaps requires sustained training, needs-based deployment, and formal recognition of field epidemiology as a distinct professional role within the public health system.

## 1 Introduction

Emerging and re-emerging infectious diseases remain a persistent threat to global health security (1). In the context of rapid ecological and societal change, the emergence of new pathogens is widely considered inevitable (2, 3). The predominantly zoonotic nature of these threats highlights the importance of the human–animal–environment interface in transmission dynamics (1, 2). Urbanization, environmental change, and global mobility further accelerate their spread, increasing the risk of large-scale outbreaks (4).

In response, the International Health Regulations (IHR 2005) require countries to develop core capacities for detecting, assessing, and reporting public health threats (5). Achieving these capacities depends on a skilled field epidemiology workforce capable of supporting surveillance, risk assessment, and emergency response (6, 7). Emerging lessons from the COVID-19 pandemic have exposed critical workforce shortages, revealing that strengthening regional and national capacity in rapid outbreak investigation is vital to ensure effective disease management (8, 9). Despite this evidence, Joint External Evaluations (JEE) continue to identify field epidemiology as a priority area for sustained investment (6, 10).

Field Epidemiology Training Programs (FETPs) were established to build this capacity through a “learning by doing” approach, combining structured training with supervised field practice to develop operational competencies (11, 12). Since their expansion in the 1980s, these programs have been widely adapted to national contexts (13). Nevertheless, FETPs are often under-prioritized within health systems. Persistent challenges include chronic funding shortages, workforce gaps, and difficulties in deploying trained personnel to the areas of greatest need. Furthermore, there is a recognized need to innovate in training delivery to maintain long-term relevance.

While most FETPs follow a three-tiered model—Frontline, Intermediate, and Advanced—implementation is frequently tailored to national priorities (14). Despite this global expansion, evidence on the geographic distribution and adequacy of trained professionals remains limited, particularly in the Middle East and North Africa region (15, 16). In Tunisia, the national Field Epidemiology Training Program (T-FETP) was launched in 2018 in partnership with the Eastern Mediterranean Public Health Network (EMPHNET). It operates through two tiers: a 1-year Intermediate level targeting regional and national staff, and a three-month Frontline program (Public Health Empowerment Program—Basic Field Epidemiology, PHEP-BFE) for local-level professionals.

Given persistent financial constraints and reliance on external funding, ensuring the sustainability of the T-FETP has become a strategic priority. While several cohorts have been trained since its inception, the program’s adequacy in meeting national requirements and ensuring equitable geographic coverage across all 24 governorates remains insufficiently documented. This study aims to assess the distribution of the field epidemiology workforce in Tunisia, quantify capacity gaps against international benchmarks, and provide evidence-based recommendations to support its institutionalization and long-term sustainability.

## 2 Materials and methods

### 2.1 Study design and population

This was a descriptive cross-sectional study based on the T-FETP graduate roster. The study population included all participants who graduated from the program at both Frontline and Intermediate levels between its inception in 2018 and the most recent cohort completing training in 2024.

### 2.2 Data source

Data were extracted from the official T-FETP roster, managed by the National Observatory of New and Emerging Diseases (ONMNE). For each participant, the database records the cohort, graduation year, program type (Intermediate or Frontline), year of birth, sex, profession, institution of origin, level of practice (local, regional, or national), and governorate of assignment.

### 2.3 Study variables

The variables analyzed included: (i) sociodemographic and professional characteristics (age, sex, profession, institution of origin); (ii) distribution by program type, cohort, and year of graduation; (iii) distribution across levels of the health system; and (iv) geographic coverage by governorate.

### 2.4 Geographic coverage analysis

Coverage was measured as the density of Intermediate-level graduates per 200,000 inhabitants. This analysis included only graduates assigned to regional or local structures; those working at the national level were excluded from the primary analysis, as national posts do not directly contribute to routine sub-national surveillance capacity. We acknowledge that national-level graduates may contribute to surge capacity during outbreaks through deployment mechanisms, but the JEE framework emphasizes demonstrated subnational capacity as the basis for assessing routine surveillance and response functions. A sensitivity analysis was conducted to assess the impact of including national-level graduates. The benchmark of one Intermediate-level graduate per 200,000 inhabitants was used, consistent with IHR 2005 monitoring frameworks and TEPHINET guidance (10, 17). Governorate-level population data were obtained from the official 2024 General Census of Population and Housing (Recensement Général de la Population et de l’Habitat 2024) datasets and demographic projections published by the National Institute of Statistics (INS) of Tunisia (18).

For each of the 24 governorates, coverage was classified into three categories: target achieved (≥1 graduate per 200,000 inhabitants); partial deficit (<1 graduate per 200,000 inhabitants, but with at least one graduate present); and no coverage (zero Intermediate-level graduates). While applying the 1:200,000 benchmark at the governorate level is a standard approach to identify sub-national disparities, we acknowledge that density estimates in low-population governorates are highly sensitive to small-number fluctuations (often based on one to three graduates). Our analysis utilizes these indicators not as absolute measures of localized coverage, but as diagnostic tools to reveal systemic territorial imbalances and the cumulative capacity deficit. The principal conclusions—a substantial national shortfall and a marked inequitable distribution—are grounded in aggregated totals and macro-structural patterns (e.g., entire densely populated governorates with zero coverage) rather than on volatile local point estimates.

### 2.5 Capacity gap analysis

The required number of Intermediate-level graduates per governorate was calculated by dividing the governorate population by 200,000 and rounding up to the nearest whole number. The gap for each governorate was then defined as the difference between this expected number and the observed number of graduates. Two metrics were derived to quantify workforce deficits. First, a simple national deficit was calculated as the net difference between the total national requirement and subnational active graduates. Second, a cumulative subnational shortfall was calculated by aggregating positive deficits only (i.e., summing the graduates needed in underserved governorates while treating surplus regions as zero). This latter metric is more operationally relevant, as epidemiological capacity in surplus areas cannot be dynamically redeployed across territorial boundaries.

### 2.6 Statistical analysis

Variables were constructed to capture both baseline profiles and temporal dynamics. Age was calculated in two ways: first, age at graduation was defined as the difference between the cohort graduation year and the participant’s year of birth; second, age in 2026 was calculated using the year of analysis as the reference year. To assess workforce aging and the potential for capacity rejuvenation over time, a cumulative median age analysis was performed. For each year from 2018 to 2026, the median age of the cumulative pool of Intermediate-level graduates (i.e., all graduates who had completed training up to that specific year) was calculated. This dynamic approach allowed for tracking the evolving age profile of the active workforce as successive cohorts entered the system, and as the workforce aged mechanically during periods without new graduates. Additionally, the proportion of graduates aged 55 years or older—corresponding to the critical seven-year window preceding the Tunisian national retirement age of 62 years—was calculated for the Intermediate-level workforce in 2022 and 2026 to quantify the impending attrition risk.

Descriptive and comparative analyses were performed using R (version 4.3.3). Continuous variables are reported as means with standard deviations (SD) or medians with interquartile ranges (IQR), as appropriate. Categorical variables are reported as frequencies and proportions. Differences in mean age between the Intermediate and Frontline groups were evaluated using Welch’s two-sample *t*-test, with statistical significance set at *p* < 0.05. Geographic density was visualized using choropleth maps generated via the ggplot2 package.

Missing data for age and professional background were explicitly examined to assess potential selection bias. Because these variables were not mandatory fields in the original training application forms, their absence reflects administrative incompleteness rather than systematic non-response related to cohort, program tier, governorate of assignment, or other graduate characteristics. While the potential for selection bias cannot be entirely excluded, this pattern suggests that the available age subsample remains broadly representative of the full graduate cohort.

### 2.7 Ethical considerations

This study used anonymized programmatic data collected as part of the ONMNE’s routine monitoring and evaluation activities. As a secondary analysis of administrative records with no identifiable personal information, formal ethical approval was not required. Data confidentiality was maintained throughout.

## 3 Results

### 3.1 Training output and cohort dynamics (2018–2024)

Between 2018 and 2024, the T-FETP trained 103 professionals across seven cohorts. Of these, 50 (48.5%) completed the Intermediate level and 53 (51.5%) the Frontline level. Intermediate cohorts averaged 12.5 participants (SD: 3.3; range: 8–15). Frontline cohorts were slightly larger, averaging 17.7 participants (SD: 2.5). Program output peaked in 2022, with 40 graduates across both levels. A three-year gap between 2019 and 2022 interrupted the Intermediate tier, and no new Intermediate cohort has been organized since 2022 (Table 1).

**Table 1:** Distribution of Tunisia-FETP graduates (2018–2024) by training program and cohort.

Program and cohort	Year	Number (n)	Percentage (%)
Intermediate FETP			
FETP cohort 1	2018	15	14.6
FETP cohort 2	2019	15	14.6
FETP cohort 3	2022	8	7.8
FETP cohort 4	2022	12	11.7
FETP subtotal	–	50	48.5
Frontline FETP (PHEP-BFE)			
PHEP-BFE cohort 1	2021	18	17.5
PHEP-BFE cohort 2	2022	20	19.4
PHEP-BFE cohort 3	2024	15	14.6
*PHEP-BFE subtotal*	–	*53*	*51.5*
Grand total	2018–2024	103	100.0

Both Cohort 3 and Cohort 4 graduated in 2022. Cohort 3 was initiated in 2021, but its graduation was delayed to early 2022 due to operational constraints and workforce deployment during the COVID-19 pandemic.

### 3.2 Participant characteristics

Regarding sex distribution, the majority of the participants were female, representing 75.7% ( n=78 ) of the total, while 24.3% ( n=25 ) were male. Complete age data were available for a subset of 50 graduates (Intermediate: *n* = 28; Frontline: *n* = 22), upon which the following dynamic analysis is based.

**Figure 1:**
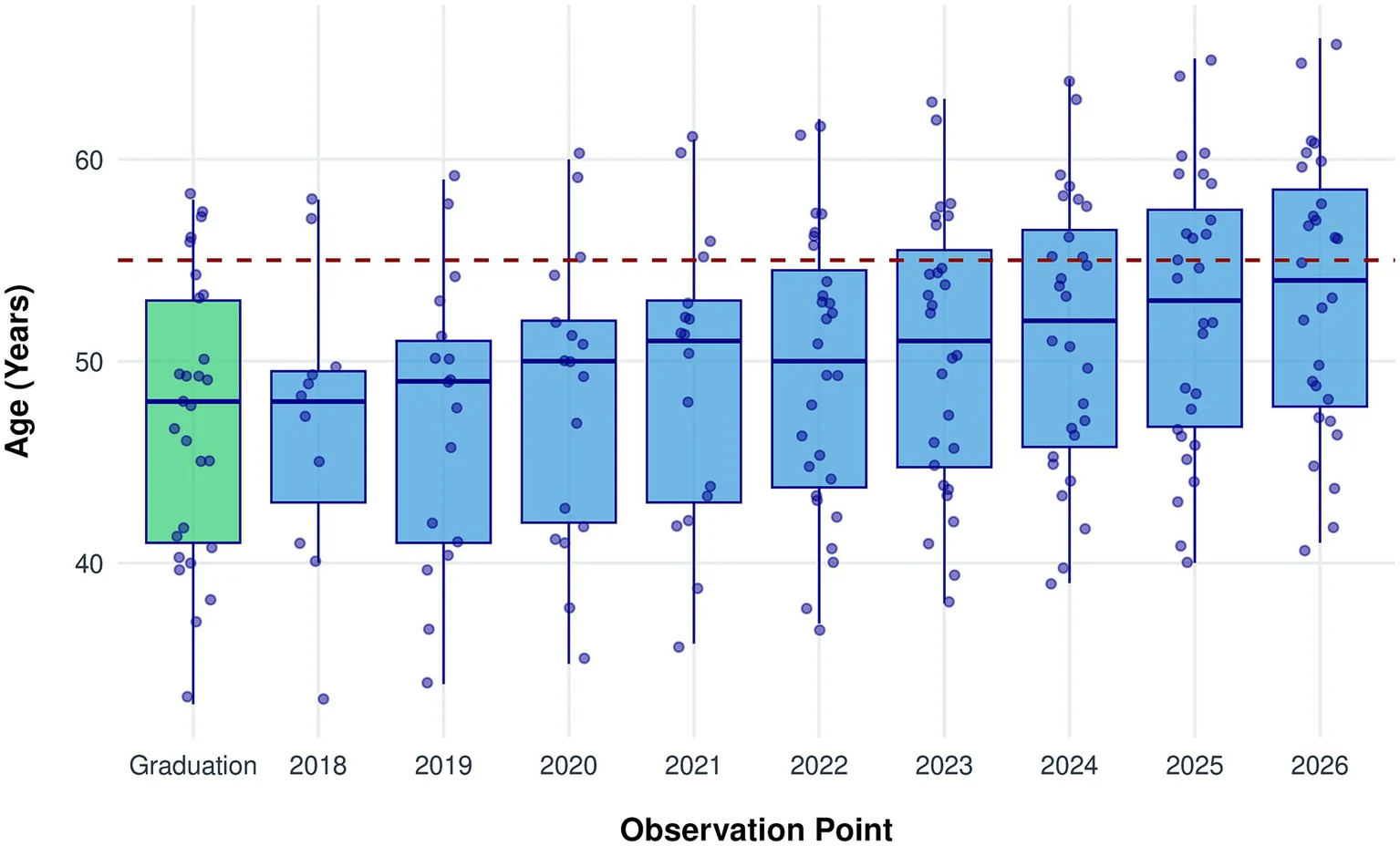
Cumulative age evolution of the FETP-Intermediate active workforce (2018–2026). Boxplots illustrate the baseline age distribution at graduation (green box, left) compared to the cumulative active graduate pool tracked at each annual observation point from 2018 to 2026 (blue boxes). Based on the subset of 28 graduates with complete age data, the median age (indicated by the bold horizontal lines) remained stable at 48 years from 2018 to 2021, then rose to 50 years in 2022 and reached 54 years in 2026. The horizontal dashed line marks the 55-year threshold—the seven-year window preceding the Tunisian national retirement age of 62 years—highlighting that the proportion of the workforce approaching retirement doubled from 25.0 to 50.0% during the 2022–2026 period of mechanical aging.

For the Intermediate FETP program, the baseline analysis of the program’s intake revealed a mature cohort profile, with a median age at graduation of 48 years (IQR: 41–53). The dynamic tracking of the cumulative active workforce pool from 2018 to 2026 demonstrated a steady shifting of the age distribution over time (Figure 1). In 2018, the initial active pool (Cohort 1) exhibited a median age of 48 years. Following the graduation of consecutive cohorts, the cumulative pool reached its maximum size of 28 graduates by late 2022. Notably, the median age of this cumulative pool remained stable at 48 years between 2018 and 2021, before rising to 50 years in 2022. Between 2022 and 2026, with no new Intermediate cohorts graduating, the existing workforce underwent mechanical aging, pushing the median age to 54 years by 2026. Consequently, the proportion of this trained epidemiological workforce nearing retirement age (aged 55 years or older) escalated sharply from 25.0% in 2022 to 50.0% in 2026.

Regarding the Frontline PHEP-BFE program, the workforce exhibited a younger profile compared to the Intermediate track, with a median age at graduation of 44 years (IQR: 38–48). Unlike the Intermediate track, the Frontline program continued to inject new competencies into the health system through 2024. By 2026, the median age of this cumulative Frontline cohort rose to 48.5 years, with 18.2% of the graduates reaching or exceeding 55 years of age.

Professional background data were available for 95 of the 103 graduates (92.2%). Physicians made up the majority at 70.5% (*n* = 67), distributed across both tiers (Intermediate: *n* = 38; Frontline: *n* = 29). Paramedical staff accounted for 14.7% (*n* = 14), all trained through the Frontline program. Veterinarians represented 9.5% (*n* = 9), exclusively through the Intermediate tier. Medical residents made up the remaining 5.3% (*n* = 5; Intermediate: *n* = 3, Frontline: *n* = 2).

### 3.3 Institutional affiliation and administrative level

Graduates came from all three levels of the Tunisian health system. The majority were from the regional level (52.4%; *n* = 54), followed by the local level (24.3%; *n* = 25) and the national level (23.3%; *n* = 24). At the regional level, 51 participants were affiliated with Regional Health Directorates, working in epidemiological surveillance, public health programs, or basic health services. Both program tiers were represented (Intermediate: n = 22; Frontline: *n* = 29). A small number came from regional agricultural offices (*n* = 1) and regional hospitals (*n* = 2). Local-level participants were based in health districts or frontline settings such as district hospitals and basic health centers. The Frontline program predominated at this level (*n* = 16 vs. *n* = 9 for Intermediate). These graduates were primarily involved in detecting and reporting public health events. National-level graduates came from central institutions, mainly the Ministry of Health (*n* = 18), the Ministry of Agriculture (*n* = 5), and the military health sector (*n* = 1). Key structures included the ONMNE, the Strategic Health Operations Center, and various technical directorates. The Intermediate program was concentrated at the national and regional levels (*n* = 16 and *n* = 25 respectively), while the Frontline program was more evenly spread across regional and local levels (*n* = 29 and *n* = 16) (Table 2).

**Table 2:** Distribution of Tunisia-FETP graduates (2018–2024) by administrative level and institution of origin.

Level/institution	FETP (intermediate)	PHEP-BFE (frontline)	Total (n)	%
National level				
Ministry of health (central)	11	7	18	17.5
Ministry of agriculture	5	0	5	4.9
Military health	0	1	1	1.0
*National subtotal*	*16*	*8*	*24*	*23.3*
Regional level				
Regional health directorate	22	29	51	49.5
Regional agricultural development office	1	0	1	1.0
Hospital facility	2	0	2	1.9
*Regional subtotal*	*25*	*29*	*54*	*52.4*
Local level (health districts)	9	16	25	24.3
Grand total	50	53	103	100.0

### 3.4 Coverage and density of FETP-intermediate graduates

Based on all 50 Intermediate-level graduates and a national population of 11,972,169, the overall density was 0.83 per 200,000 inhabitants. In a sensitivity analysis including national-level graduates, the zero-coverage status of key urban hubs and the core sub-national structural deficits remained unchanged. Restricting the analysis to the 34 graduates working at the regional and local levels, this density decreased to 0.57 per 200,000 inhabitants.

**Figure 2:**
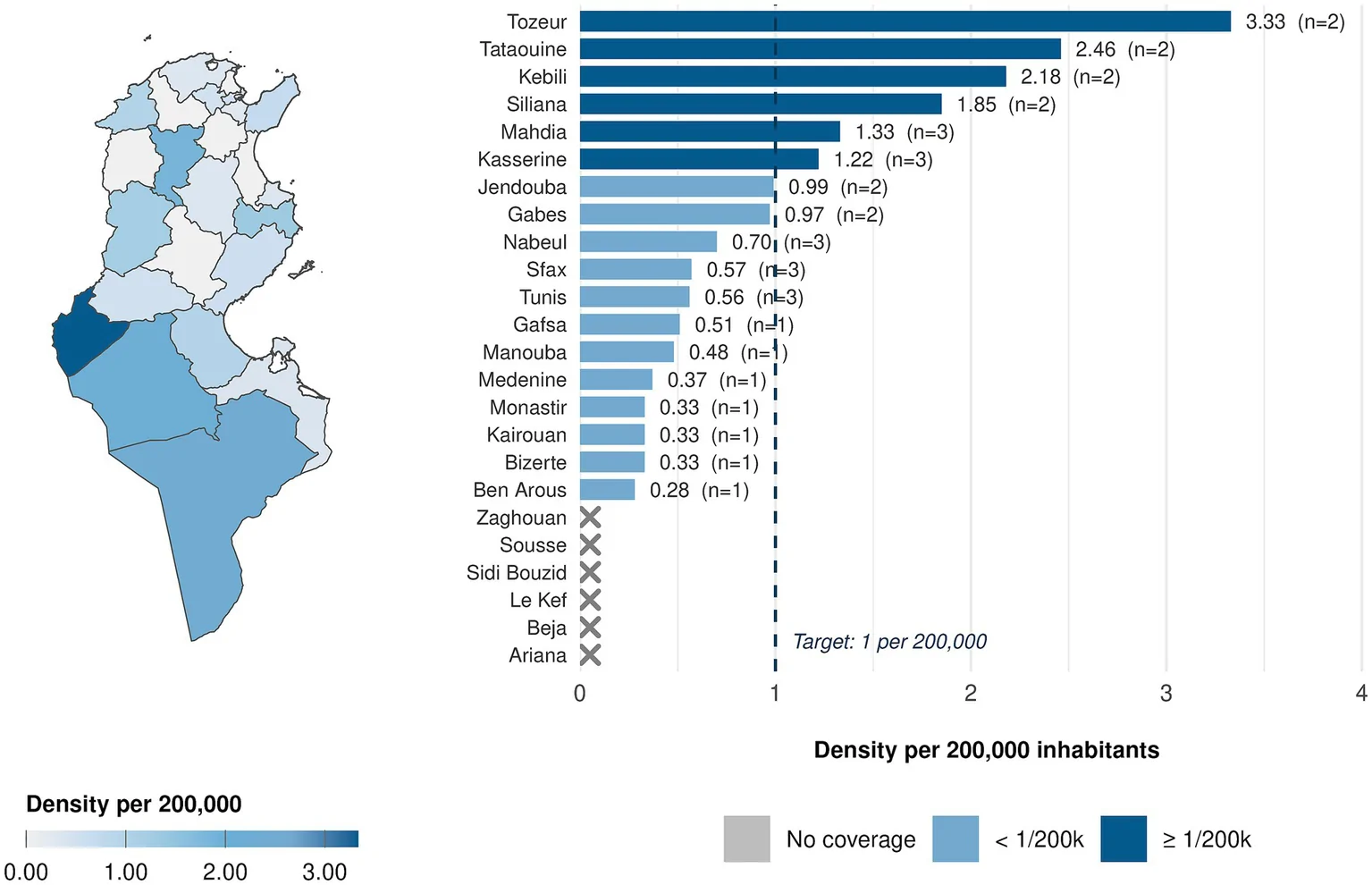
Geographic distribution and density per 200,000 inhabitants of T-FETP graduates (2018–2024) by governorate. The choropleth map (left) illustrates the distribution of Intermediate-level graduates across the 24 governorates. The horizontal bar chart (right) ranks governorates by their density, with a vertical dashed line indicating the national target of 1 graduate per 200,000 inhabitants. Data labels display the density followed by the absolute number of graduates (*n*) for each governorate. Governorates marked with an “X” indicate areas with no Intermediate-level FETP coverage.

Across the 24 governorates, six (25%) achieved the target of at least one Intermediate-level graduate per 200,000 inhabitants: Tozeur (3.33), Tataouine (2.46), Kebili (2.18), Siliana (1.85), Mahdia (1.33), and Kasserine (1.22). Twelve governorates (50%) reached partial coverage but remained below the threshold. The remaining six (25%)—Sousse, Ariana, Sidi Bouzid, Béja, Le Kef, and Zaghouan—had no Intermediate-level coverage (Figure 2).

Densities varied substantially across governorates. Higher values were observed in sparsely populated areas such as Tozeur and Tataouine, whereas more populous governorates tended to have lower densities. For example, Tunis (0.56) and Sfax (0.57) remained below the threshold despite having three graduates each. Notably, Sousse, with over 762,000 inhabitants, had no Intermediate-level graduates (Figure 2).

It should be noted that coverage classifications in low-population governorates are based on small absolute numbers (e.g., *n* = 2 in Tozeur and Tataouine). However, the overarching findings—namely, a substantial national capacity gap and the complete absence of Intermediate-level epidemiologists in key urban hubs—reflect macro-structural deficits that remain robust to workforce fluctuations in any single location.

### 3.5 Capacity gap analysis of the FETP-intermediate workforce

Meeting the international benchmark of one FETP-Intermediate graduate per 200,000 inhabitants across all 24 governorates would require a total of 74 professionals. With only 34 currently active at subnational levels, the cumulative shortfall across underserved governorates is 43 graduates. This cumulative shortfall (43) exceeds the simple national deficit of 40 (74–34) because the 34 subnational graduates are not optimally distributed; some governorates have surpluses that cannot be redeployed to cover gaps elsewhere.

The analysis of unmet needs reveals three distinct levels of vulnerability. While six governorates met or exceeded their requirements, 12 are in a state of partial deficit. The most substantial quantitative gaps in this category are concentrated in the coastal and metropolitan hubs of Sfax, Tunis, and Bizerte, each requiring at least three additional graduates to reach minimum capacity. The most critical shortfall, however, exists in the six governorates with zero Intermediate-level coverage. Sousse and Ariana represent the highest priority for recruitment, with each requiring a minimum of four additional epidemiologists.

## 4 Discussion

Over its first 6 years (2018–2024), the T-FETP has contributed to the development of a substantial national epidemiological workforce, with 103 professionals trained across seven cohorts (50 Intermediate and 53 Frontline) and combined program coverage extending to all 24 of the country’s governorates. That is a real achievement for a program operating in a resource-constrained environment. It is also, frankly, not enough. The national shortfall stands at 43 Intermediate-level graduates, three quarters of governorates do not meet the international benchmark, and the program has produced no new Intermediate cohort since 2022. The picture is one of genuine progress alongside significant structural fragility. Comparisons with other regional programs help put these findings in context. The Jordan FETP, often cited as a regional benchmark, went through a similar expansion phase before reaching the institutional stability it now enjoys (19). Tunisia is at an earlier stage of that trajectory. The dual-tier structure—Intermediate plus Frontline—aligns well with TEPHINET’s recommended architecture, which aims to build competencies at every level of the health system (20, 21).

Women make up 75.7% of graduates, which is consistent with the broader feminization of health professions in Tunisia (22). This contrasts with findings from Khader et al., who reported a male predominance among FETP trainees in the Eastern Mediterranean Region and Bangladesh (16).

The age profile of the trained workforce represents perhaps the most pressing concern for the sustainability of Tunisia’s epidemiological capacity. Rather than a stable demographic baseline, our analysis reveals a critical temporal shift: within just 4 years (2022–2026), the absence of newly graduated Intermediate cohorts resulted in a mechanical aging of the active workforce, with the median age rising from 50 to 54 years.

The most alarming implication is that 50.0% of Intermediate-level field epidemiologists are now aged 55 years or older—meaning that half of this highly specialized workforce is within 7 years of the national retirement age (62 years in Tunisia). This situation mirrors, yet surpasses in severity, challenges documented in other national programs. For instance, in the Philippines, the median age of practicing field epidemiologists reached 52 years, with nearly 20% expected to retire within 5 years (23). In Tunisia, the median age has already reached 54 years, with 50% of the workforce approaching imminent retirement—more than double the proportion observed in the Philippines.

In Tunisia, this impending demographic shift threatens to create a significant depletion of institutional knowledge, leadership, and operational mentorship capacity if a deliberate succession plan is not urgently institutionalized. Furthermore, while the Frontline tier successfully attracted a younger professional profile (median age at graduation: 44 years) and continued to inject new competencies through 2024, the significant age differential between the two programs (7.8 years, *p* = 0.002) underscores a structural bottleneck. The Intermediate program is not attracting or retaining younger professionals at the rate required to ensure workforce rejuvenation, highlighting the urgent need for formal career pathways, competitive compensation, and clearly defined roles within the national surveillance system.

Physicians dominate the workforce at 70.5%, which mirrors patterns seen in other FETPs where medical training is the primary entry point (24). Paramedical staff are present exclusively at the Frontline level, while veterinarians are represented only in the Intermediate tier. This latter finding is significant: a 9.5% veterinary participation rate is a meaningful step toward a genuine One Health approach. However, given that zoonotic risks such as brucellosis and rabies are endemic in Tunisia (25, 26), the absence of veterinarians at the Frontline level remains a critical gap. This lack of local-level integration may hinder early detection at the human–animal interface, particularly in rural agricultural areas (3). Recent international frameworks such as the COHFE competencies explicitly call for multisectoral field epidemiology training (27, 28), and the T-FETP’s inclusion of veterinarians at the Intermediate level is a concrete step in that direction. Joint training of human and animal health professionals fosters shared practices and strengthens multisectoral collaboration, which is essential in settings with persistent zoonotic risks (29).

To assess national capacity, we applied the benchmark of one field epidemiologist per 200,000 inhabitants, as defined in the Joint External Evaluation (JEE) framework (10). Due to the scarcity of parameters estimating the real number of field epidemiologists needed, this rate was used as the required baseline for Intermediate-level graduates. This threshold, derived from earlier epidemiological capacity assessments and adapted to low- and middle-income settings, applies specifically to Intermediate and Advanced levels (17). In contrast, Frontline capacity is better assessed through administrative coverage, typically defined as the presence of at least one trained surveillance officer per district (30).

Using this framework, Tunisia reaches a national density of 0.83 Intermediate epidemiologists per 200,000 inhabitants, reflecting substantial progress since the 2016 JEE, which identified the absence of a formal FETP as a critical gap (31). However, this aggregate figure masks important structural imbalances. When focusing on territorial operational capacity and excluding centrally assigned staff, the density decreases to 0.57 per 200,000. This approach is consistent with the JEE 2022 emphasis on demonstrated subnational capacity rather than nominal national availability (10). It shows that nearly one-third of the workforce is concentrated at the central level.

Subnational disparities are pronounced. Only six of the 24 governorates meet the recommended threshold, while six have no Intermediate-level epidemiologist at all. The distribution appears inversely related to epidemiological risk. Less populated regions meet or exceed the benchmark, whereas densely populated urban centers such as Sousse and Ariana remain underserved. These gaps represent critical vulnerabilities, as delayed detection in high-density and high-mobility areas can accelerate outbreak propagation (32). This pattern is consistent with findings documented elsewhere in the region. While our data do not allow us to test the underlying mechanisms, previous work has suggested that workforce distribution often reflects institutional dynamics—such as training placement patterns and career advancement opportunities—rather than population health needs (3). Similarly, as noted by Flint et al., a recurring challenge in FETP evaluations is the poor utilization of graduates by senior management, often due to limited understanding of their potential contribution to the health system (33). In the Tunisian context, it is plausible that similar dynamics may be at play, though this remains a hypothesis to be tested in future implementation research.

These findings highlight a key limitation of the 1:200,000 benchmark. While useful for international comparison, it captures potential capacity rather than effective system performance (33, 34). Consequently, national averages may conceal structural weaknesses, where a high headcount of trained professionals fails to translate into operational resilience due to a lack of institutional integration and formal career pathways (33, 35).

The sustainability of the system is further challenged by structural constraints. Interruptions in training cycles and the absence of Intermediate cohorts since 2022 have slowed workforce production. At the current pace, the program is unlikely to close the identified gap of 43 additional epidemiologists within a reasonable timeframe. Besides the poor access to training for health professionals in rural and other underserved areas, the rapid evolution of education and training systems through the use of new technologies—which are often scarce or unavailable—constitutes another crucial challenge for the T-FETP. Furthermore, the absence of a formal deployment mechanism limits how well the workforce matches epidemiological needs. In addition, the advanced age profile of Intermediate graduates introduces a foreseeable attrition risk. The lack of formal recognition of the ‘field epidemiologist’ role within the civil service may contribute to underutilization of trained professionals. While this study does not directly assess utilization patterns, this phenomenon has been documented in other settings as “brain waste” (36) and represents a plausible hypothesis for the observed capacity-utilization gap in Tunisia. In a workforce where nearly three-quarters of graduates are women, this structural gap also raises concerns about the retention and optimal use of a predominantly female public health workforce. These challenges are consistent with findings from other middle-income settings, where limited institutionalization and fragmented workforce policies constrain the impact of FETP programs (24). They also reflect a broader systemic pattern observed in Tunisia, where technical capacities may coexist with weaker operational planning and implementation, as highlighted by international assessments such as the Global Health Security Index (37). Finally, the program depends heavily on external funding. This is a vulnerability shared by many FETPs in low- and middle-income settings (36), but it is particularly acute when domestic financing mechanisms are absent. Interruptions like the 2019–2022 gap may reflect, at least in part, the instability of project-based funding cycles.

Addressing these issues requires a shift from a training-centered approach to a system-oriented strategy. This includes strengthening institutional anchoring, ensuring sustainable financing, and aligning workforce deployment with national needs. Increasing training volume and frequency will be necessary to offset attrition and close coverage gaps. Establishing formal career pathways is equally critical to retain trained professionals and support progression from Frontline to Intermediate roles. Finally, workforce planning should move beyond a purely demographic approach and incorporate needs-based criteria, including population density, mobility patterns, and environmental exposure (38). In the wake of the COVID-19 pandemic and ongoing health system reforms, the institutionalization of the T-FETP is no longer a technical option but a strategic imperative for national health security.

This study has several limitations. It is a static snapshot of the roster at a single point in time, with no data on workforce dynamics such as retirements, reassignments, or departures from the public sector. The actual number of graduates still active in field epidemiology roles may therefore be lower than reported. Data completeness was limited for key variables, including age and professional background, restricting subgroup analyses. These fields were not mandatory in the training application forms, resulting in administrative missingness rather than systematic bias related to program tier or geographic assignment. In addition, the study does not assess the operational performance or competencies of graduates, nor their direct contribution to outbreak response. These dimensions remain essential for a comprehensive evaluation of program impact. Although governorate-level density estimates in low-population areas are statistically fragile and subject to local volatility, the study’s primary objectives—identifying a substantial national deficit and major regional disparities—remain robust. These conclusions are driven by aggregated data and macro-structural gaps (e.g., entire densely populated governorates lacking any Intermediate-level graduates) rather than by unstable local point estimates. This distinction is critical for accurately interpreting the findings for public health policy and workforce planning.

In conclusion, while the T-FETP has successfully established a foundational national field epidemiology workforce with comprehensive geographical coverage within 6 years, significant subnational gaps remain. The severe shortage of Intermediate-level epidemiologists, coupled with marked territorial disparities and structural constraints, underscores that scaling up training volumes alone is insufficient. Addressing these challenges requires transitioning to a system-oriented approach that integrates institutional anchoring, sustained training cycles, needs-based deployment, and formal public health career pathways. Ensuring these systemic improvements is critical to translate training outputs into sustainable, long-term surveillance and response capacity across all levels of the Tunisian health system.

## Data Availability

The raw data supporting the conclusions of this article will be made available by the authors, without undue reservation.
